# Searching for genetic modulators of the phenotypic heterogeneity in Brugada syndrome

**DOI:** 10.1371/journal.pone.0263469

**Published:** 2022-03-01

**Authors:** Laura Martínez-Campelo, Raquel Cruz, Alejandro Blanco-Verea, Isabel Moscoso, Eva Ramos-Luis, Ricardo Lage, María Álvarez-Barredo, María Sabater-Molina, Pablo Peñafiel-Verdú, Juan Jiménez-Jáimez, Moisés Rodríguez-Mañero, María Brion

**Affiliations:** 1 Cardiovascular Genetics, Santiago de Compostela Health Research Institute, Santiago de Compostela, Spain; 2 Genomic Medicine Group, Universidade de Santiago de Compostela, Santiago de Compostela, Spain; 3 CIBER of Rare Diseases, Carlos III Health Institute, Madrid, Spain; 4 Cardiovascular CIBER, Carlos III Health Institute, Madrid, Spain; 5 Cardiology Group, Center for Research in Molecular Medicine and Chronic Diseases (CIMUS), Universidade de Santiago de Compostela, Santiago de Compostela, Spain; 6 Cardiogenetics Laboratory, Murcian Institute for Biosanitary Research, Cardiology Service, Virgen de la Arrixaca University Clinical Hospital, Murcia, Spain; 7 Arrhythmia Unit, Virgen de las Nieves University Hospital, Granada, Spain; 8 Cardiology Service, Santiago de Compostela University Hospital, Santiago de Compostela, Spain; 9 Family Heart Disease Unit, Cardiology Service, Santiago de Compostela University Hospital, Santiago de Compostela, Spain; University of Tampere, FINLAND

## Abstract

In Brugada syndrome, even within the same family where all affected individuals share the same mutation, phenotypic variation is prominent, with variable penetrance and expressivity, presenting different degrees of involvement. It is difficult to establish a direct correlation between genotype and phenotype to predict prognosis in complications and risk of sudden death. The factors that modulate this inter- and intra-familial phenotypic variability remain to be determined. With the intention of testing whether other genetic factors, in addition to the causal mutation in *SCN5A*, may have a modulating effect on the Brugada phenotype and the risk of sudden death, we have studied 8 families with a causal variant in *SCN5A* with at least two affected individuals, one of whom has suffered cardiac arrest or sudden death. Whole exome sequencing was performed looking for additional variants that modify the phenotype and allow us to predict a better or worse prognosis for the evolution of the disease. The results did not show any clear genetic modifier; nevertheless, highlight the possible implication of the cholesterol and fibrosis pathways, as well as the circadian rhythm, as possible modulators of Brugada syndrome phenotype.

## 1. Introduction

Brugada syndrome (BrS) is an inherited arrhythmogenic disease, with a prevalence of 1/2000, that may lead to sudden cardiac death in young adults with structurally normal hearts. To date, more than 350 rare variants associated with BrS have been described within the *SCN5A* coding region, which encodes the cardiac sodium channel, representing 20–25% of clinically diagnosed BrS cases. Almost 150 additional variants in other sodium, calcium or potassium channels genes are proposed to be causative of BrS, but together these explain no more than 10% of cases and they are thus considered minor genes [1, 2]. Although BrS is considered a genetic disease, the molecular mechanism remains unknown in 70–85% of clinically confirmed cases. This is because many patients follow a non-Mendelian inheritance, present mutations in non-coding regions or in unknown genes or even copy number variations (CNVs) [3, 4].

In recent years, evidence has increased about the role of CNVs in SCN5A gene as a cause of Brugada syndrome. So far, about 10 CNVs potentially associated with the disease have been described in this gene [5–7]. Recently, Jimmy Juang et al. described a copy number deletion of *GSTM3* in BrS patients, associated with reduced I_Na_ and higher rates of syncope, suggesting a novel potential genetic modifier/risk predictor for the development of BrS [3].

It has been observed that BrS-associated variants show incomplete penetrance and highly variable expressivity among carriers. That phenotypic variability might be the result of additional modifiers of channel behaviour, such as other genetic variation and alterations in transcription, RNA processing, translation, post-translational modifications, and protein degradation [8]. Most of the genetic studies carried out until a few years ago have focused on coding variants, such as the pioneering study by Marangoni et al, where they describe that the clinical differences between BrS and long QT syndrome have an additional genetic cause even in the presence of the same heterozygous SCN5A variant [9]. However, more recent genome-wide association studies (GWAS) reveal that most disease-associated variants are found in non-coding regions, especially in cis-regulatory regions. [10]. For instance, common variants in *SCN5A*, *SCN10A*, *HEY2* and, more recently, in *IRX3*, located in non-coding regions, have emerged as potential modulators of the disease by affecting different regulatory mechanisms [11, 12]. Otherwise, common variants in the *SCN5A* promoter, and especially those affecting transcription factor binding sites (TFBSs), are associated with alterations in the transcriptional activity of *SCN5A* [13–15]. Additionally, a recent report also showed that *IRX5* and *GATA4* act synergistically to activate the *SCN5A* promoter in human heart [16].

On the other hand, mutations in mitochondrial tRNA genes and a specific mitochondrial DNA allelic combination have also been suggested as potentially involved in BrS [17].

All of these findings highlight the impact of non-coding variants, which are binding targets for miRNAs, in altering miRNA-target cross-talk. In recent years it has been shown that microRNAs such as miR-24, miR-98, miR-106, miR-200, miR-219, and miR-1270 regulate *SCN5A* expression, thus contributing to the high risk of subsequent cardiac arrythmias [18–21].

In general, common variants could be exacerbating or attenuating the disease manifestation alongside a primary genetic defect, which is usually described in a coding region, but these variants can also be responsible for the disease phenotype in the absence of coding variants [22]. As new studies appear it is becoming more evident that both coding and non-coding regions play a fundamental role in the pathophysiology of BrS [23].

Even though *SCN5A* is the principal contributing factor to BrS, all the observations aforementioned support the growing hypothesis that BrS pathogenesis follows an oligogenic or multigenic model [24, 25]. Based on this model, BrS may not be caused by a single rare variant, but rather by the presence of multiple susceptibility variants acting synergistically through one or more mechanistic pathways while being influenced by the environment [8].

In recent years, cardiovascular studies have been focused on personalized risk assessment and to determine the most optimal therapy for an individual. This is especially tricky in BrS, especially in the cases of multiple variants within the same individual, which can have a combined pathological effect. For this, it is essential to understand the genetics of this syndrome and a good genotype-phenotype correlation, which is the objective of this study. In order to elucidate whether other genetic variants in coding regions are affecting the phenotypic expression of individuals with BrS and a causal genetic variant in *SCN5A*, we have carried out a whole exome sequencing study in families with several affected individuals and variable severity.

## 2. Results

### 2.1. Analysis of uncommon variants among individuals with discordant phenotype within each family

**Fig 1:**
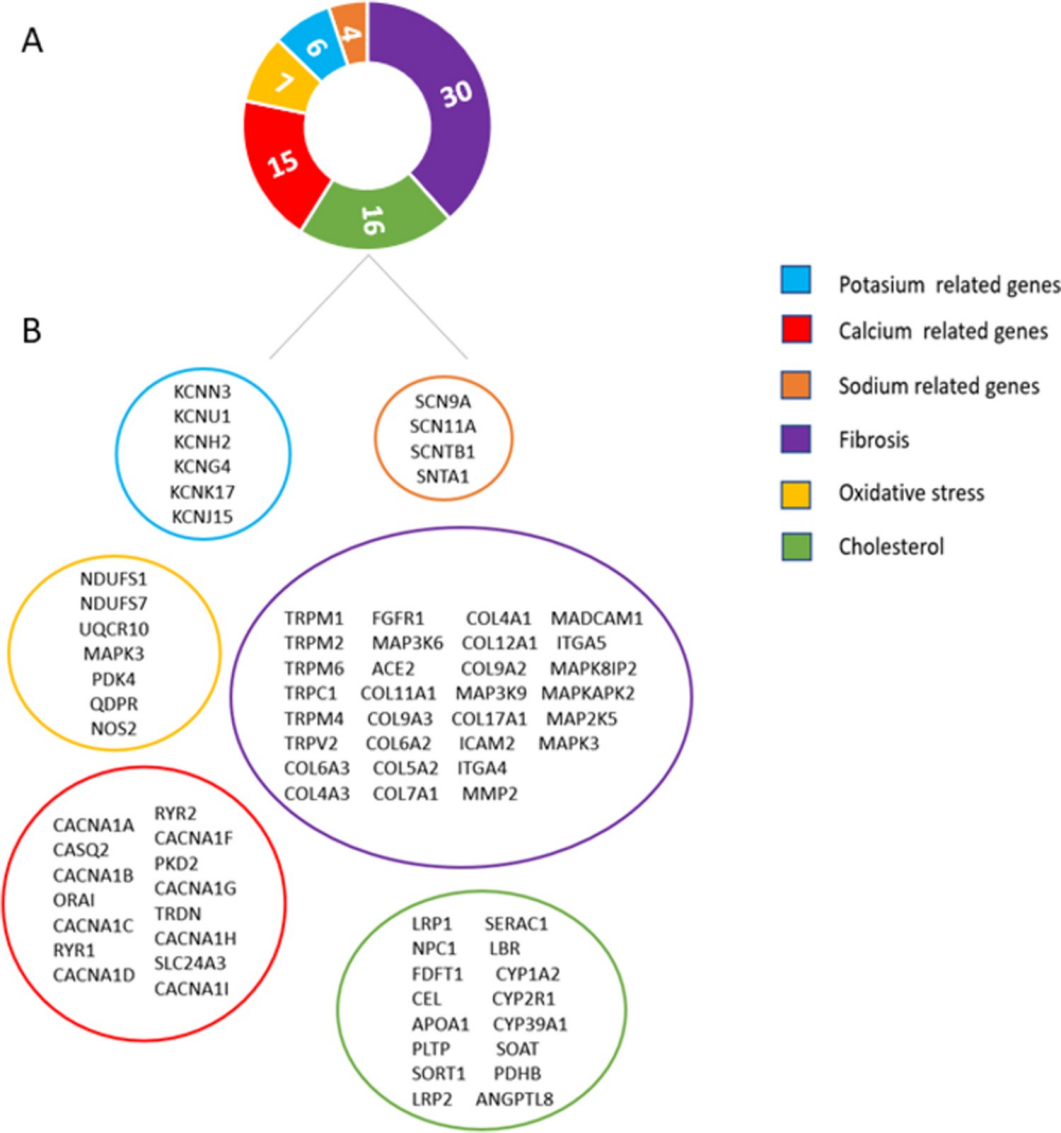
Main altered pathways. (A) Number of genes per pathway. (B) Genes of the pathway.

After the variant prioritization process according to the probability of being involved in BrS, we observed an average of 7.4 ± 2.26 discordant variants between the severe and mild individuals of each family. None of the prioritized variants recurs among several families and none of them presents sufficient evidence to be considered as a modifier of the Brugada phenotype. However, if we look at genes and pathways rather than individual variants, we found that many genes showing discordant variants within each family could be grouped in 6 pathways, which may highlight the role of these pathways in the syndrome (Fig 1).

Classification of genes, using biological process terms, showed that ion channels genes were the most significant class of genes with uncommon variants (31/84 genes; 36.9%); therefore, all analysed families harbour at least one ion channel gene with uncommon variation between relatives with discordant phenotypes. Specifically, highlights the calcium channel, and by extension it signalling pathway, with up to 8 different subunits of the channel and other 7 related genes with uncommon variants in 7 of the 8 families. These findings seem to indicate a key role of this cation in BrS, as Monasky et al. previously pointed out [26]. Additionally, sodium and potassium channel genes also showed uncommon variants. Different genes that encode TRP channels, collagen genes, adhesion molecules, integrins, matrix metalloproteinase, MAP kinases, fibroblast growth factor receptor and angiotensin were also represented, suggesting the influence of fibrosis processes in this disease. In the same way, several genes that participate in oxidative stress, which plays a key role in fibrosis, have been found.

Finally, an enrichment of cholesterol related genes has also been seen. In particular, LDL receptors and genes involved in biosynthesis, trafficking and metabolism of cholesterol.

It is interesting to note that other genes previously associated with BrS such as *SLMAP*, *AKAP9* and *ANK2* have also been found.

### 2.2. Load of variant by phenotypic groups

A total variant count (excluding the causal mutation) was also performed in the *SCN5A* gene, the main gene associated with Brugada Syndrome and carrier of the pathogenic or likely pathogenic variant in these patients. Making express reference to the mean number of variants between both phenotypic groups, we found 5 ± 1.07 variants in the severe and 5.06 ± 0.26 variants in mild group. Of these 5 variants, in general, 3 or 4 were exonic and 1 or 2 intronic, only two samples had 1 splicing variant.

Taking into account the total number of variants in Brugada genes, no difference was found, with an average of 213.37 ± 32.44 variants / severe subject versus 210.86 ± 34.21 variants / mild subject (p = 0.86). Looking for the type of variant, the percentage of non-synonymous mutations is also very similar in both groups (23.02% severe versus 23.49% mild).

Considering the whole exome of each individual, there was no significant difference in the burden of functional rare variants between the severe (216 ± 45.7 variants / subject) and the mild BrS phenotype (208.4 ± 22.7 / subject) (p = 0.73). Specifically, the percentage of variants that cause truncated proteins was 12.38% in the severe group and 10.84% in the mild phenotype group. Regarding the *in silico* prediction of pathogenicity, it has been observed a mean of 28.4 ± 8.56 variants considered harmful with 4 predictors in the severe group compared to 31 ± 3.08 variants observed in the mild group (p = 0.54).

### 2.3. Gene-base analysis

**Fig 2:**
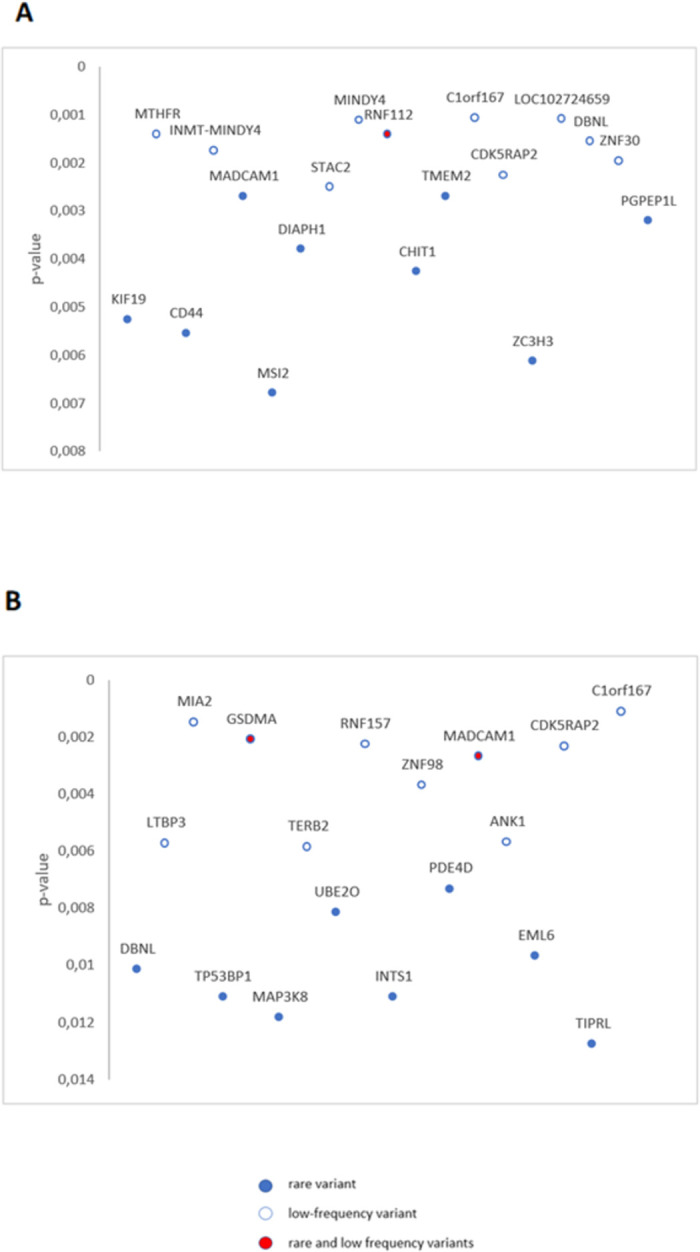
Top genes from KMgene analysis carried out with (A) all the rare and low-frequency variants (B) rare and low-frequency functional variants. The genes that came out in the two analysis are represented with the lowest p-value.

The gene-base analysis carried out with all the rare variants, low-frequency variants, rare functional variants and low-frequency functional variants did not show any gene with significant signal after Bonferroni test. Fig 2 shows the top genes, reflecting the ten most significant p-value of the different approaches for each gene, adjusted for age and sex as covariates.

### 2.4. Discriminant analysis of principal components

Discriminant analysis was performed considering only first-degree relatives and also considering all the individuals; however, the discrimination between the group of severe and mild phenotype in both analysis was not good enough. The analysis assigned up to 3 individuals of severe phenotype as mild. Furthermore, the coefficient of the discriminant function showed quite low values and no marker stood out.

## 3. Discussion

To date, BrS genetic bases have been incompletely elucidated. In spite of >20 genes associated with this disorder, their real impact in pathogenesis is still controversial, especially, the genotype-phenotype correlation. Despite of harbouring a pathogenic/likely pathogenic *SCN5A* variant, the clinical manifestations are extremely varied, ranging from a lifetime of asymptomatic disease to sudden death in the young, which makes decision-making difficult. In the present study, more than a specific gene, we found possible pathways that could be modifying the phenotypic severity of BrS.

A variety of ion channels, like voltage-gated Na^+^, Ca^2+^ and K^+^ channels, have been shown to be regulated by changes in the level of membrane cholesterol. The most common effect is cholesterol-induced decrease in channel activity that may include decrease in the open probability, unitary conductance and/or the number of active channels on the membrane [27, 28]. In some cases, changes in membrane cholesterol affect biophysical properties of the channel such as the voltage dependence of channel activation or inactivation [29]. For example, plasma hypercholesterolemia resulted in decreased current density of voltage-gated Na^+^ currents, slower recovery from inactivation, and more negative potential for inactivation of the sodium inward currents [30]. In addition, maintaining membrane cholesterol level is required for coupling ion channels to signalling cascades. In this sense, the cholesterol related genes that we found differently mutated in our severe and mild phenotype population could be altering the levels of cholesterol in the cell membrane causing changes in the properties of the sodium channel, as well as in the calcium or potassium channel, worsening the phenotype, which would be a new modifier pathway in this disease not indicated until now.

We also found altered several genes that participate in the fibrosis process. Structural abnormalities have been reported previously in BrS patients [31, 32]. Nademanee et al. showed an increased collagen content in the right ventricular outflow tract (RVOT) that shows epicardial surface and intramyocardial fibrosis, in addition to diminished gap junction protein expression. This fibrosis was associated with conduction delay in the RVOT [33, 34]. Our results go in the same direction as these articles and seem to indicate that despite being an arrhythmogenic disease there are certain structural abnormalities in this syndrome.

One important mechanism involved in fibrosis is oxidative stress. In this sense, we found differentially mutated 2 NADH: ubiquinone oxidoreductase subunits, 1 ubiquinol-cytochrome C reductase subunit, 1 *MAPK*, *PDK4* and *QDPR* gene, which would be involved in an overproduction of reactive oxygen species and damage in the mitochondrial respiratory chain, as pointed out previously by various authors [35, 36].

It seems relevant to us to comment that in one family we found a rare variant in the *CLOCK* gene present in the individual with severe phenotype and absent in his mild relative. Although this variant was classified as uncertain significance according to the ACMG recommendations [37], *CLOCK* is a transcriptional factor that, together with its heterodimer *BMAL1*, form the core of the circadian clock, which control the circadian rhythm through a transcriptional/translation feedback loop [38]. It has been seen that up to 10% of the cardiac transcriptome is controlled by the local cardiac clock [39]. As a result, key processes in the heart (including ion channel remodeling and electrical excitability, signal transduction and metabolism) vary in a circadian manner [40].

*SCN5A*, the major contributor to Na^+^ current in ventricular myocytes, is under the temporally regulation of the cardiac clock and followed a circadian pattern of mRNA expression (increased expression during dark period) [41, 42]. Several studies showed that loss of *CLOCK-BMAL1* resulted in a loss of the circadian expression of *SCN5A*, with the consequent reduction of Nav1.5 levels and a decrease I_Na_ in the presumptive dark phase [41, 43]. The carrier of the *CLOCK* variant, who suffered 5 previous syncopes, has a loss-of-function *SCN5A* mutation with a presumptive reduction of I_Na_ current. The additional *CLOCK* variant could aggravate this phenotype by increasing the reduction of the I_Na_ current, causing a high susceptibility to arrhythmias. Additionally, fatal ventricular arrhythmias in BrS often occurs during sleep at night, which could be explained in part by this temporary reduction of the I_Na_ current in dark period. Everything of the above mentioned suggests that circadian clock could modulate the susceptibility to arrhythmias in this syndrome, which has not been seen until now.

Thinking about a possible accumulation of rare variants in *SCN5A* and in other genes associated with BrS, the simple count of these variants did not show significant differences between the group of patients with a severe form of the disease compared with the group with a mild manifestation. Also, no differences were found in the individual count of variants between individuals with severe and mild involvement within each family.

The genetic approach presented in this study is based on exome sequencing, so it is focused on the search on coding or adjacent regions, losing information of intronic regions, possible regulatory and structural variation information. Although we did not find phenotype-modulating genetic variants on their own, our study supports the previous articles by suggesting the contribution of several genes to establish the phenotype of an individual and emphasizes the difficulty of achieving a definitive molecular diagnosis. We highlight the possible implication of the cholesterol and fibrosis pathways, as well as the circadian rhythm, as possible modulators of BrS phenotype, but this data are not conclusive, it is necessary to carry out larger sample size studies and functional experiments to clarify it.

## 4. Materials and methods

### 4.1. Sample collection and selection

The cases were selected in the cardiology services of the Clinical University Hospital of Santiago, the Clinical University Hospital of Virgen de la Arrixaca of Murcia and University Hospital Virgen de las Nieves of Granada, based on the following criteria: families with at least 2 individuals with a clinical diagnosis of BrS confirmed by spontaneous or induced electrocardiographic pattern 1, carriers of pathogenic or likely pathogenic genetic variant in *SCN5A* gene and discordant phenotype, with one of the relatives with severe clinical involvement and the others asymptomatic or with mild symptoms. Severe clinical involvement was considered in those patients with previous cardiorespiratory arrest, syncope or with positive electrophysiological study for arrhythmia inducibility.

In total, 23 individuals of European origin (8 with severe phenotype and 15 with none or mild BrS phenotype) corresponding to 8 families were collected (Table 1). There were 11 women and 12 men with a mean age of 54 years old.

**Table 1:** Study population description.

Family	Individual	Sex	Age	Diagnostic basal ECG	Drug challence test	Electrophysiological study	Syncope history	Cardiac arrest history	ICD	Phenotype	SCN5A genetic variant
**1**	1a	male	40	+	+	+	-	-	+	severe	NM_198056:c.5227G>A,p.(Gly1743Arg)
	1b	female	45	-	+	-	-	-	-	mild	
	1c	male	54	+	NA	-	-	-	-	mild	
	1d	male	67	-	-	-	-	-	-	mild	
**2**	2a	male	80	+	NA	NA	+	-	+	severe	NM_198056:c.5227G>A,p.(Gly1743Arg)
	2b	female	41	+	NA	-	-	-	-	mild	
**3**	3a	male	38	+	-	-	-	-	-	mild	NM_198056:c.5227G>A,p.(Gly1743Arg)
	3b	female	45	+	NA	NA	+	-	+	severe	
	3c	female	54	-	NA	NA	-	-	-	mild	
	3d	female	63	-	-	NA	-	-	-	mild	
**4**	4a	female	20	+	+	NA	+	-	-	severe	NM_198056:c.5227G>A,p.(Gly1743Arg)
	4b	female	75	-	+	NA	-	-	-	mild	
	4c	male	43	-	NA	NA	-	-	-	mild	
**5**	5a	male	77	-	NA	NA	-	-	+	mild	NM_198056:c.4426C>T,p.(Gln1476*)
	5b	male	65	-	NC	-	-	-	-	mild	
	5c	male	76	-	+	NA	+	-	+	severe	
**6**	6a	male	48	-	+	-	+	+	+	severe	NM_198056:c.535C>T,p.(Arg179*)
	6b	female	25	-	+	-	-	-	-	mild	
**7**	7a	female	64	-	-	+	+	+	+	severe	NM_198056:c.5445dupT,p.(Asp1816*)
	7b	male	34	-	+	-	-	-	-	mild	
	7c	female	63	-	-	-	-	-	-	mild	
**8**	8a	female	57	NA	NA	NA	-	+	-	severe	NM_198056:c.2302A>G,p.(Ile768Val)
	8b	male	69	+	NA	NA	-	-	-	mild	

Abbreviations: + (positive),—(negative), NA (not available), NC (not conclusive).

The study was conducted according to the guidelines of the Declaration of Helsinki, and approved by the Santiago-Lugo regional delegation of the Research Ethics Committee of Galicia (protocol code: 2017/197 and date of approval: 26 June 2017). Written informed consent was obtained from all subjects involved in the study.

### 4.2. Exome sequencing

After DNA extraction we checked DNA integrity by agarose gel electrophoresis and DNA quantity by Invitrogen^TM^ Qubit^TM^ from Thermo Fisher Scientific. The process of capturing the regions of interest was carried out by xGen Exome Research Panel v2 from IDT that targets only the coding sequences of human coding genes in the RefSeq 109 database. Sequencing was performed in NextSeq 500 from Illumina, and variant calling and annotation was performed by means of: BWA, GATK, Pindel, Picard, BEDTools, SAMTools, ExomeDepth and ANNOVAR, using the GRCh37/hg19 reference genome [44]. Sequence data has been deposited at the European Genome-phenome Archive (EGA), which is hosted by the EBI and the CRG, under accession number EGAS00001005848.

### 4.3. Variant analysis in individuals with divergent phenotypes

The variants obtained in the sequencing process are subjected to a prioritization process according to their probability of being involved in BrS. This analysis was performed with the R software, based on the following criteria:

- Frequency in European (non-Finish) population of the GnomAD consortium database lower than the prevalence of BrS.
- Variants located in exonic or splicing regions.
- Variants that do not cause synonymous changes in the protein.
- The number of times that variant appears in the internal database is less than 5 to CNVs and less than 10 to SNV.
- Variants in a list of 274 candidate genes selected according to the etiopathogenesis of BrS.

With the intention of identifying the genetic factors that may be contributing to phenotypic variability in each family, among the prioritized variants, we selected the uncommon variants between the individual with severe phenotype and the relative with milder phenotype.

### 4.4. Genetic variant load comparisons between groups with divergent phenotypes

Two genetic variant load comparisons between the group of patients with severe phenotype and the group of patients with milder phenotypes was performed: 1) considering only the variants in the *SCN5A* gene and 2) considering the variants in a list of 43 genes previously associated with BrS (Table 2).

**Table 2:** Genes previously associated with Brugada syndrome.

ABCC9	HCN4	KCNT1	SCNN1A
AKAP9	HEY2	LRRC10	SCN2B
ANK2	KCNAB2	PLN	SLMAP
CACNA1C	KCNB2	PKP2	SEMA3A
CACNA2D1	KCND2	RYR2	TRPM4
CACNB2	KCND3	RANGRF	TBX5
CASQ2	KCNE3	SCN5A	TKT
DSG2	KCNE5	SCN10A	TTN
DSP	KCNH2	SCN1B	XIRP1
FGF12	KCNJ8	SCN3B	XIRP2
GPD1L	KCNJ16	SCN4A	

Within each family we also looked for rare functional uncommon variants between first degree relatives with severe and mild phenotype. For this purpose, rare variants are considered to be those with a maximum frequency of 0.01, and functional means those variants that produce non-synonymous changes in the protein or truncating variants, considered as such, frameshift, stopgain and canonic splicing (≤ ± 2pb) variants. We used four *in silico* predictors for pathogenicity: SIFT (available in https://sift.bii.a-star.edu.sg/), Polyphen2 (available in http://genetics.bwh.harvard.edu/pph2/), MutationTaster (available in http://www.mutationtaster.org/), and PROVEAN (available in http://provean.jcvi.org/genome_submit_2.php?species=human).

### 4.5. Gene-base analysis

We use the R package KMgene for performing gene-based association tests taking into account the relationship in families, and using kernel machine (KM) regression under a generalized linear mixed model framework to identify genes that differentiate the two phenotypic groups. Gene-based tests are able to identify weak individual signals by combining the effects of variants in the same gene, and greatly reduce the number of multiple testing. Although this method can test both rare and common variants, in the present study, rare variants (MAF < 0.01), low-frequency variants (MAF < 0.1), functional rare variants and functional low-frequency variants were separately collapsed in each gene, assuming equal weight for all markers and adjusting for age and sex covariates.

### 4.6. Analysis of genetically structured populations

To identify variants that contribute to the differentiation between the severe and the mild individuals in each family, we performed a cluster-based discriminant analysis of principal components (DAPC), that is a multivariate approach carried out with Adegenet library, available for R software. We carry out this analysis from the list of uncommon variants among first-degree relatives of the section 4.4.
